# Syntaxin 1A Gene Is Negatively Regulated in a Cell/Tissue Specific Manner by YY1 Transcription Factor, Which Binds to the −183 to −137 Promoter Region Together with Gene Silencing Factors Including Histone Deacetylase

**DOI:** 10.3390/biom11020146

**Published:** 2021-01-23

**Authors:** Takahiro Nakayama, Toshiyuki Fukutomi, Yasuo Terao, Kimio Akagawa

**Affiliations:** 1 Department of Medical Physiology, Kyorin University School of Medicine, Tokyo 181-8611, Japan; yasuo.terao@gmail.com (Y.T.); akagawak@ks.kyorin-u.ac.jp (K.A.); 2 Department of Pharmacology and Toxicology, Kyorin University School of Medicine, Tokyo 181-8611, Japan; fukutomi@ks.kyorin-u.ac.jp

**Keywords:** SNARE, non-neuronal, histone deacetylase, gene silencing, heterochromatin, neurodevelopmental disorder

## Abstract

The HPC-1/syntaxin 1A (*Stx1a*) gene, which is involved in synaptic transmission and neurodevelopmental disorders, is a TATA-less gene with several transcription start sites. It is activated by the binding of Sp1 and acetylated histone H3 to the −204 to +2 core promoter region (CPR) in neuronal cell/tissue. Furthermore, it is depressed by the association of class 1 histone deacetylases (HDACs) to *Stx1a*–CPR in non-neuronal cell/tissue. To further clarify the factors characterizing *Stx1a* gene silencing in non-neuronal cell/tissue not expressing *Stx1a*, we attempted to identify the promoter region forming DNA–protein complex only in non-neuronal cells. Electrophoresis mobility shift assays (EMSA) demonstrated that the −183 to −137 OL2 promoter region forms DNA–protein complex only in non-neuronal fetal rat skin keratinocyte (FRSK) cells which do not express *Stx1a*. Furthermore, the Yin-Yang 1 (YY1) transcription factor binds to the −183 to −137 promoter region of *Stx1a* in FRSK cells, as shown by competitive EMSA and supershift assay. Chromatin immunoprecipitation assay revealed that YY1 in vivo associates to *Stx1a*–CPR in cell/tissue not expressing *Stx1a* and that trichostatin A treatment in FRSK cells decreases the high-level association of YY1 to *Stx1a*-CPR in default. Reporter assay indicated that YY1 negatively regulates *Stx1a* transcription. Finally, mass spectrometry analysis showed that gene silencing factors, including HDAC1, associate onto the −183 to −137 promoter region together with YY1. The current study is the first to report that *Stx1a* transcription is negatively regulated in a cell/tissue-specific manner by YY1 transcription factor, which binds to the −183 to −137 promoter region together with gene silencing factors, including HDAC.

## 1. Introduction

HPC-1/syntaxin 1A (*Stx1a*) was first discovered as a gene exhibiting neuron-specific expression and is a key acceptor molecule of a complex called soluble N-ethylmaleimide-sensitive fusion protein attachment protein receptor (SNARE), which is involved in presynaptic vesicle exocytosis in the central nervous system (CNS) [1,2,3,4]. The hemizygous deletion of this gene causes an autosomal dominant neurodevelopmental disorder, Williams syndrome, which is characterized by a unique behavioral and cognitive profile, mild-to-moderate intellectual disability, multi linguistic ability, attention-deficit hyperactivity and poor visuo-spatial recognition [5,6,7]. We previously reported that the *Stx1a* gene is a TATA-less gene with multiple transcription start sites and is activated by the binding of Sp1 and acetylated histone H3 (AcH3) to the −204 to +2 core promoter region (CPR) in neuronal cell and tissue [8]. We also found that the neuron-specific expression of this gene is determined by gene silencing via the binding of class 1 histone deacetylases (HDACs), such as HDAC1, HDAC2 and HDAC8, to *Stx1a*–CPR in non-neuronal cell and tissue [8,9]. However, the detailed transcription mechanism involved in *Stx1a* gene silencing is unclear.

To further clarify the factors characterizing *Stx1a* gene silencing in non-neuronal cell and tissue, in the present study, we first attempted to identify the promoter region forming DNA–protein complex only in non-neuronal cells by electrophoresis mobility shift assays (EMSA). EMSA demonstrated that DNA–protein complex is formed on the −183 to −137 OL2 region of *Stx1a*–CPR only in non-neuronal cells such as fetal rat skin keratinocyte (FRSK) and fibroblast 3Y1 not expressing *Stx1a*. Competitive EMSA and supershift assay also revealed that the DNA–protein complex formed on the −183 to −137 region consists of the Yin-Yang 1 (YY1) transcription factor in FRSK cells. Furthermore, the association of YY1 to the *Stx1a*–CPR in vivo was examined by chromatin immunoprecipitation (ChIP) assay. Results showed that YY1 associates to *Stx1a*–CPR in cell/tissue not expressing *Stx1a* and that trichostatin A (TSA) treatment in FRSK cells decreases the high-level association of YY1 to the *Stx1a*–CPR in default. YY1 belongs to the GLI-Kruppel class of zinc finger transcription factors and is a ubiquitously distributed transcription factor that positively or negatively regulates a diverse number of gene promoters. YY1 is implicated in histone modification via interaction with various proteins, including histone acetyltransferases and HDACs, to activate or repress gene promoters [10,11,12,13]. We then examined the effect of YY1 on *Stx1a* transcription activity by conducting a luciferase reporter assay. Results showed that YY1 negatively regulates *Stx1a* transcription. Finally, we identified proteins that bind to the −183 to −137 promoter region by mass spectrometry analysis. Results suggest that gene silencing factors, including HDAC1, associate to the −183 to −137 promoter region together with YY1. In the present study, we demonstrated that *Stx1a* gene transcription is negatively regulated in a cell-/tissue-specific manner by the YY1 transcription factor and that YY1 binds to the −183 to −137 promoter region together with factors participating in gene silencing, such as HDACs.

## 2. Materials and Methods

### 2.1. Cell Culture

Cell lines of fetal rat skin keratinocyte (FRSK), rat fibroblast 3Y1, rat adrenal pheochromocytoma PC12 and human neuroblast IMR32 were purchased from HSRRB (Osaka, Japan). *Drosophila melanogaster* SL2 cell line was purchased from ATCC (Manassas, VA, USA). Cells were maintained in RPMI medium or Schneider’s drosophila medium supplemented with 10% (*v/v*) fetal calf serum (FCS), streptomycin and penicillin.

### 2.2. Electrophoresis Mobility Shift Assays (EMSA) and Supershift Assay

Preparation of nuclear extracts, EMSA and supershift assays were performed as previously described [8]. The sequence positions from the translation initiation site of used OL1–8 are as follows. OL1 (−210 to −178), OL2 (−183 to −137), OL3 (−140 to −118), OL4 (−120 to −88), OL5 (−87 to −69), OL6 (−71 to −55), OL7 (−54 to −26) and OL8 (−25 to +2). Each annealed ds-oligonucleotide fill-in labeled with [α-32P] dCTP and exo-Klenow fragment was incubated with poly(dI-dC) and nuclear extract in EMSA buffer (20 mM HEPES, pH 7.9, 25 mM KCl, 2 mM MgCl_2_, 0.1 mM EDTA, 0.5 mM DTT, 2 mM spermidine, 0.1 mg/mL bovine serum albumin and 10% glycerol). For competition assays, 0–125 pmol of cold oligonucleotides was added to the reaction mixtures and incubated before probe addition. For supershift assay, polyclonal antibodies to SP1 (Active Motif, Carlsbad, CA, USA), SP3 and SP4 (Santa Cruz, CA, USA) and monoclonal antibodies to YY1 (H10) and NFκB (F-6) (Santa Cruz) were used. Antibody (4 µg) was incubated with nuclear extract in EMSA buffer at 4 °C for 16 h before the addition of the OL2 probe. EMSA reaction mixtures were loaded and separated on a native 3–5% polyacrylamide gels containing 0.5× Tris borate–EDTA buffer. Dried gels were exposed to BASS-2000 imaging plate (IP) film (FUJIFILM, Tokyo, Japan). 

### 2.3. Chromatin Immunoprecipitation (ChIP) Assay 

ChIP assay was adapted from reference [8] with modifications. In brief, cells treated for 48 h with or without 1 µM TSA were fixed with 1% formaldehyde and resuspended in swelling buffer (5 mM 1,4-Piperazinediethanesulfonic acid (PIPES), pH 8.0, 8.5 mM KCl, 0.5% NP-40 and 0.1% protease inhibitors (PI) cocktail). For the preparation of fixed tissue, rat brain and lung tissues profusely fixed with 1% paraformaldehyde in PBS were post-fixed and resuspended in swelling buffer. Nuclei were collected from homogenized cells or tissues and sonicated to an average length of 200–500 bp. The precleared chromatin solution was incubated with control antibody (Jackson Laboratory, Sacramento, CA, USA), AcH3 antibody (FUJIFILM Wako Pure Chemical, Osaka, Japan) or YY1 antibody and then reacted with protein G-beads blocked with sheared herring sperm DNA and bovine serum albumin (BSA). Following two washing steps, immune complexes were eluted with an elution buffer (50 mM NaHCO_3_ and 1% sodium dodecyl sulfate (SDS)). The supernatant from the reaction lacking a primary antibody was saved as total input chromatin and processed alongside the eluted immunoprecipitates, beginning at the cross-link reversal step. After the cross-links were reversed with NaCl and RNaseA and incubated with proteinase K, the samples were precipitated and analyzed by PCR. PCR was carried out with primers rStx1ag F−284 to −255 (5′-CTG GGG CAG AGG AGG CAG CAG GGT CTC TGG-3′) and rStx1a R+30 to +11 (5′-CAT ACA AAA CTC CTA AAT TC-3′). Cycling conditions were as follows: 95 °C for 3 min, followed by 35 cycles of 95.5 °C for 30 s, 50 °C for 30 s and 72 °C for 40 s. PCR products were run on a 1.5% agarose gel and visualized by *SYBR Green* staining.

### 2.4. Transient Cotransfection Assays and Luciferase Reporter Gene Assays

Luciferase reporter assay was carried out in accordance with our previous report [8]. For reporter assays in *Drosophila melanogaster* SL2 cells, pPac-mock, pPac-YY1, and/or pPac-Sp1 vectors were co-transfected with phRG-TK and pGL3-CPR (−204 to +2) reporter vectors into cells by using FuGENE 6 (Roche, Basel, Switzerland). For reporter assays in PC12 cells, pcDNA3-mock or pcDNA3-YY1 vector was co-transfected with phRG-TK and pGL3-CPR (−204 to +2) reporter vectors. At 48 h after transfection, cells were lysed in reporter passive lysis buffer (Promega, WI, USA). Luciferase activity in cell lysates was measured by a luminometer (Turner Designs, San Jose, CA, USA). Relative luciferase units were evaluated after normalization against the phRG activity of each sample. Fold change was expressed as relative luciferase unit with pPac or pcDNA expression plasmid/relative luciferase unit with a mock vector.

### 2.5. DNA Affinity Purification of Transcription Factor

The isolation of DNA–protein complexes based on streptavidin and biotin interaction was adapted from reference [14,15] with modifications. Magnosphere MS300 (TaKaRa, Shiga, Japan) streptavidin magnetic beads (1 mg) were incubated for 30 min at 25 °C with 1 nmol biotinylated OL2 DNA in 0.4 ml of binding buffer (5 mM Tris-HCl, pH 7.5, 1 M NaCl, 0.5 mM EDTA and 0.05% Tween20). Nuclear extracts (50–500 µg) were precleared for 1 h at 4 °C with 1 mg of streptavidin magnetic beads and incubated for 16 h at 4 °C with the biotinylated OL2 DNA–streptavidin magnetic beads in 0.2 mL of binding buffer (10 mM HEPES-KOH, pH 7.6, 50 mM KCl, 5 mM MgCl_2_, 1 mM EDTA, 5 mM DTT, 1 mM spermidine, 0.1 µg/µl poly(dI-dC), 0.01% BSA, 10% glycerol and 0.1% PI cocktail). The reaction beads were washed with binding buffer and eluted by boiling in Laemmli sample buffer. Eluted samples were subjected to 5% SDS–PAGE gel electrophoresis. Gels were stained with Coomassie blue and then prepared for mass spectrometry analyses.

### 2.6. Mass Spectrometry Analyses

All protein bands were excised from a Coomassie blue-stained SDS-PAGE gel and treated with trypsin for 16 h at 37 °C. Digested proteins were desalted and concentrated with StageTips [16]. Tryptic peptides were run through a mass spectrometer system (LTQ-Orbitrap, Velos mass spectrometer, Thermo scientific, MA, USA) coupled with a direct nano-liquid chromatography system (DiNA; KYA Technologies, Tokyo, Japan). MASCOT 2.4 (Matrix Science, Boston, MA, USA) search engine was used to access the UniprotKB/Swiss-prot or NCBInr database. Proteins associated with the −183 to −137 OL2 promoter region were extracted by the following criteria: unique peptide number (≥2); molecular function, such as DNA nucleotide binding; with cellular component localized to the nucleus or chromosome; and molecular weight.

### 2.7. Statistical Analysis

Data were expressed as means ± SE and analyzed using *t*-test or one-way analysis of variance. Differences at *p* values less than or equal to 0.05 were considered significant.

## 3. Results

### 3.1. YY1 Transcription Factor Associates with the −183 to −137 OL2 Region of the Stx1a Core Promoter Region in Non-Neuronal Cells Which Do Not Express Stx1a

**Figure 1:**
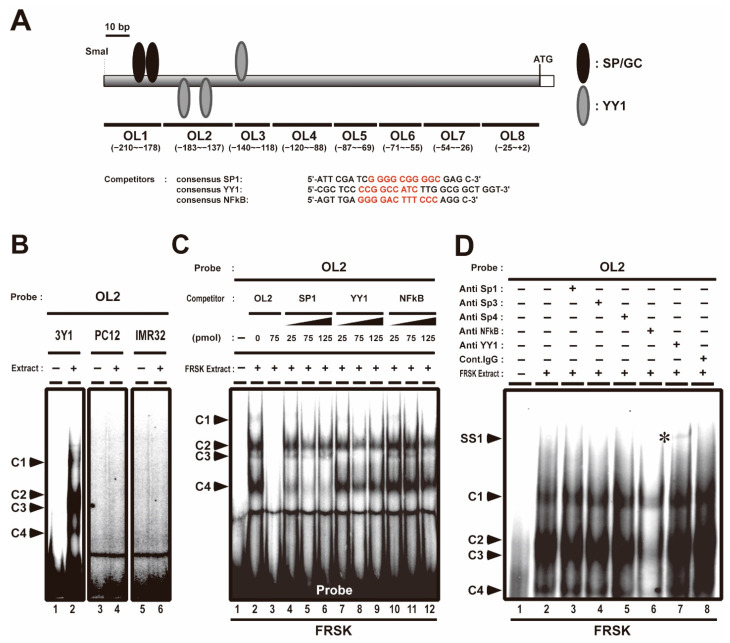
Competitive electrophoresis mobility shift assays (EMSA) and supershift assays with OL2 (−183 to −137) region in the core-promoter region (CPR) of *Stx1a*. (**A**): Scheme of OL2 probe and competitors (consensus OL) used with competitive electrophoresis mobility shift assays (EMSA). The consensus sequences of SP, YY1 and NFκB are indicated in red. (**B**): EMSA with OL2 for binding to nuclear extract of 3Y1 fibroblast cells, PC12 adrenal pheochromocytoma cells and IMR32 neuroblast cells. Arrowheads indicate shift-bands by DNA–protein complexes (C1, C2, C3 and C4). (**C**): Competitive EMSA with OL2 for binding to nuclear extract of fetal rat skin keratinocyte (FRSK) cells. Arrowheads indicate shift-bands by DNA–protein complexes (C1, C2, C3 and C4). (**D**): Supershift assay of the OL2–protein complex in FRSK cells. Nuclear extract from FRSK cells was incubated with antibodies for Sp1, Sp3, Sp4, NFκB and YY1 before the addition of the OL2 probe. Arrowheads indicate the shift-band by DNA–protein and DNA–protein–antibody complex. The supershift bands (SS1) by DNA–protein–antibody complex are indicated with asterisks.

We previously reported that the association of Sp1 and AcH3 with *Stx1a* −204 to +2 CPR (accession no. D10392) promotes *Stx1a* transcription in neuronal cell/tissue; furthermore, we showed that the association of class 1 HDACs, such as HDAC1, HDAC2 and HDAC8, with *Stx1a*–CPR negatively regulates *Stx1a* transcription in non-neuronal cell/tissue [8]. *Stx1a*–CPR includes two SP/GC sites (the −202 to −193 and −190 to −181 regions, Figure 1A), to which Sp1 associates with in neuronal cells [8]. EMSA was carried out using oligonucleotide probes (OL1–8) covering the −204 to +2 region of the CPR in non-neuronal cells such as fetal rat skin keratinocyte FRSK and rat fibroblast 3Y1 and neuronal cells such as rat adrenal pheochromocytoma PC12 and human neuroblast IMR32 to study the binding properties of *Stx1a*–CPR correlating to the *Stx1a* expression. Among these oligonucleotide probes, we found that the OL2 probe, including the −183 to −137 region of the *Stx1a*-CPR, forms four complexes (C1, C2, C3 and C4 in Figure 1B,C, lane 2) only in nuclear extract from non-neuronal 3Y1 and FRSK cells not expressing *Stx1a.* On the other hand, the OL2 probe forms no complexes (Figure 1B, lane 4 and 6) in nuclear extract from neuronal PC12 and IMR32 cells. The formation of all DNA–protein complexes (C1 to C4) disappeared upon the addition of an OL2 cold oligonucleotide competitor (Figure 1C, lane 3), indicating that these protein-forming complexes specifically bind to the −183 to −137 OL2 region of *Stx1a*–CPR. Furthermore, competitive EMSA with a cold oligonucleotide with a consensus sequence of SP, YY1 or NFκB demonstrated that the formation of the C4 DNA–protein complex decreased upon the addition of a cold oligonucleotide competitor with an SP consensus sequence (Figure 1C, lanes 4 to 6); furthermore, the formation of the C3 DNA–protein complex decreased upon the addition of the cold oligonucleotide competitor with a YY1 consensus sequence (Figure 1C, lanes 7 to 9). The formation of the C1 DNA–protein complex decreased upon the addition of any cold oligonucleotide competitor with a consensus sequence of SP, YY1 or NFκB (Figure 1C, lanes 4 to 12). The cold oligonucleotide competitors with a consensus sequence of SP, YY1 or NFκB did not affect the formation of the C2 DNA–protein complex (Figure 1C, lanes 4 to 12).

To confirm the association of these transcription factors with the −183 to −137 OL2 region of the *Stx1a*–CPR in non-neuronal cells, we next carried out a supershift assay by using an antibody against the Sp family, NFκB and YY1. After the FRSK nuclear extract was preincubated with YY1 antibody, a slow migrating supershift band was detected (SS1 in Figure 1D, lane 7, marked with an asterisk). However, no supershift bands were observed in supershift assays using Sp1, Sp3, Sp4 and NFκB antibodies (Figure 1D, lane 2 to 6), which did not support the result of the decreased C1 and C4 complex formation by the consensus oligonucleotide of SP, YY1 and NFκB (Figure 1C). This result suggested that the decreased C1 and C4 complexes may have been due to the weak affinity of the protein to the OL2 probe or another protein recognizing the compatible consensus sequence of SP, YY1 and NFκB. 

These finding indicated that the YY1 transcription factor associates with the −183 to −137 OL2 region of the *Stx1a*–CPR in non-neuronal FRSK cells which do not express *Stx1a*.

### 3.2. YY1 Associates with Stx1a–CPR in Cells and Tissues not Expressing Stx1a

**Figure 2:**
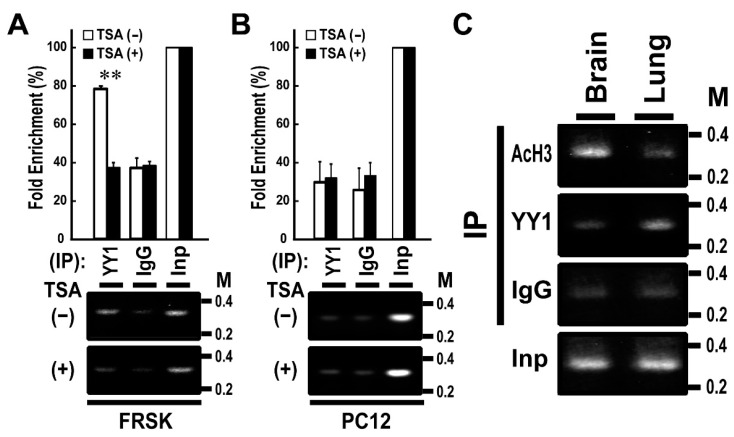
Chromatin immunoprecipitation (ChIP) assays using YY1 antibody in FRSK cells and tissues. (**A**,**B**): ChIP assays using YY1 antibody in FRSK (**A**) and PC12 (**B**) cells treated with TSA (+) or untreated (−). Chromatin DNA isolated by immunoprecipitation was amplified using primers for the *Stx1a-CPR*. The data shown are representative images. Values indicate means ± SE (*n* = 3). ** *p* < 0.01 compared to the untreated control. (**C**) ChIP assays using AcH3 and YY1 antibodies in tissues of brain or lung. Chromatin DNA isolated by immunoprecipitation was amplified using primers for the *Stx1a*–*CPR*.

We previously reported that *Stx1a* expression is induced via the association of AcH3 with *Stx1a*–CPR in non-neuronal FRSK cells by an HDAC inhibitor, TSA [8]. To further verify the above in vitro binding data, we performed ChIP assay by using YY1 antibody and chromatin extracted from FRSK cells treated with or without TSA. The ChIP assay demonstrated that the TSA treatment of FRSK cells decreased the high-level association of YY1 with the *Stx1a*–CPR in default (*p* < 0.01 to TSA untreated; *t*-test; Figure 2A). By contrast, the TSA treatment of PC12 cells did not change the association of YY1 with *Stx1a*–*CPR*, which remained at low level in default (Figure 2B). 

To study the relationship between the association of YY1 with the *Stx1a*–CPR and the tissue specificity of *Stx1a* expression, we then carried out a ChIP assay of two tissue types: rat brain tissue expressing *Stx1a* and rat lung tissue not expressing *Stx1a* [8]. The ChIP assay demonstrated that AcH3 but not YY1 binds to *Stx1a*–CPR in brain tissue, whereas YY1 but not AcH3 binds to *Stx1a*–CPR in lung tissue (Figure 2C). These results indicated that YY1 associates with *Stx1a*–CPR in non-neuronal cell/tissue not expressing the *Stx1a* gene.

### 3.3. YY1 Negatively Regulates the Stx1a Transcription

**Figure 3:**
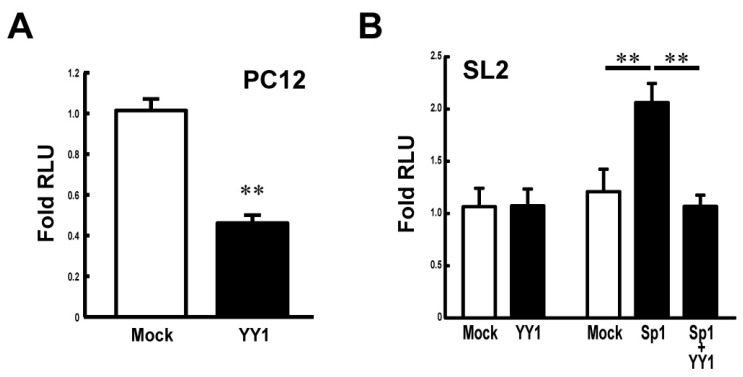
Effect of YY1 on the *Stx1a* transcription activity. (**A**): Reporter assay in PC12 cells overexpressing YY1. Reporter activity was measured in PC12 cells co-transfected with pGL3–CPR (−204 to +2) reporter vector and pcDNA–YY1 expression vector. Values represent means ± SE (*n* = 9) from three independent experiments. ** *p* < 0.01 compared to the pcDNA-vector mock control. (**B**): Reporter assay in drosophila SL2 cells overexpressing YY1 and Sp1. Reporter activity was measured in SL2 cells transfected with reporter vector pGL3–CPR (−204 to +2) and expression vectors of pPac-Sp1 and -YY1. Values represent means ± SE (*n* = 9) from three independent experiments. ** *p* < 0.01 compared to the pPac-vector mock control.

To study the effect of YY1 on the *Stx1a* transcription, the luciferase reporter assay was carried out in PC12 cells co-transfected with pcDNA-YY1 vector. Figure 3A shows that the overexpression of YY1 in PC12 cells suppresses *Stx1a* transcription. To further examine the effect of YY1 and also in combination with Sp1, on *Stx1a* transcription, we next co-transfected plasmids encoding each of these transcription factors together with *Stx1a* promoter-luciferase reporter plasmids into SL2 *Drosophila melanogaster* cells, which lack endogenous expression of the *Drosophila* homolog of Sp1 [16]. The reporter assay demonstrated that the *Stx1a* promoter activity in SL2 cells was not changed when co-transfected with pPac-YY1 (Figure 3B). On the other hand, co-transfection assay revealed that the *Stx1a* promoter activity increased 2-fold by pPac-Sp1 (*p* < 0.01 compared with pPac-vector control) is suppressed by co-transfection of pPac-YY1 (*p* < 0.01 compared with pPac-Sp1) (Figure 3B). These results suggest that YY1 negatively regulates the *Stx1a* transcription. 

### 3.4. Gene Silencing Factors, Including Histone Deacetylase, Associates with the −183 to −137 OL2 Promoter Region

**Figure 4:**
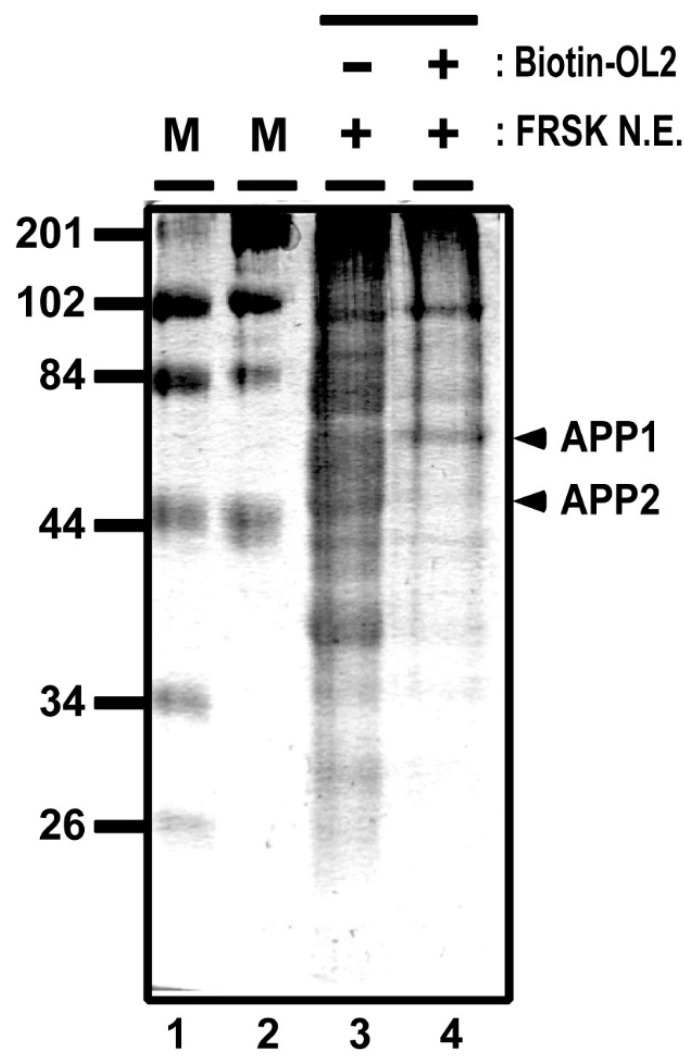
DNA affinity purification for mass spectrometry analyses. Proteins associating to the biotinylated OL2 DNA were affinity purified from FRSK nuclear extracts by avidin beads and separated with 5% sodium dodecyl-sulfate-polyacrylamide gel electrophoresis (SDS-PAGE). OL2 DNA affinity-purified protein bands (APP1 and APP2) are indicated with arrowheads.

To further identify OL2 DNA–protein complexes, we carried out mass spectrometry analysis by using proteins that were affinity purified from FRSK nuclear extracts by biotinylated OL2 DNA (see Figure 4 for a denatured gel separation image). One band of the affinity-purified proteins (Figure 4, APP1, marked with an arrowhead) extracted from the gel was measured by mass spectrometry. The mass spectrometry analysis demonstrated that APP1 may include proteins, such as non-POU domain-containing octamer-binding protein, heterogeneous nuclear ribonucleoprotein K, HDAC1, lamina-associated polypeptide 2 beta, nuclear factor 1 and heterochromatin protein 1-binding protein 3 (Table 1). In general, most of these proteins represented by HDAC1 appear to be involved in gene silencing and heterochromatin formation [17,18,19,20]. This result supports a previous study showing that class 1 HDAC, including HDAC1, only associates with *Stx1a*–CPR in non-neuronal cell/tissue not expressing *Stx1a* [8]. Furthermore, by measuring another band of affinity-purified proteins (Figure 4, APP2, marked with an arrowhead) by mass spectrometry, we confirmed that YY1 associates with the −183 to −137 OL2 promoter region (Table 2). These data suggest that gene silencing factors, including HDAC, associate with the −183 to −137 promoter region together with YY1.

## 4. Discussion

In the present study, we found that the −183 to −137 OL2 region of *Stx1a*–CPR forms DNA–protein complex only in the non-neuronal FRSK cells which do not express *Stx1a*. Furthermore, *Stx1a* transcription is negatively regulated via the binding of YY1 transcription factor to the −183 to −137 region of *Stx1a*–CPR in non-neuronal cell/tissue not expressing *Stx1a*. Moreover, the high level association of YY1 to the *Stx1a*–CPR in default in non-neuronal FRSK cells is decreased by the treatment of the HDAC inhibitor TSA. Mass spectrometry analysis also showed that YY1 binds to the −183 to −137 promoter region together with factors involved in gene silencing, such as non-POU domain-containing octamer-binding protein [18], heterogeneous nuclear ribonucleoprotein K [19], HDAC1 [20] and heterochromatin protein 1-binding protein 3 [21]. Interestingly, among these factors, class 1 HDACs, including HDAC1, are reported to associate to *Stx1a*–CPR only in non-neuronal cell/tissue not expressing *Stx1a* [8]. Furthermore, in the current study, we demonstrated that the OL2 −183 to −137 promoter region forms DNA–protein complexes only in non-neuronal FRSK cells not expressing *Stx1a*. Given that the YY1 transcription factor is well known to interact with proteins, including HDACs [11,12,13,22], the gene silencing factors, including HDACs, may associate onto the OL2 −183 to −137 promoter region of *Stx1a* via complex formation with the YY1 transcription factor. 

Taken together, these findings suggest that *Stx1a* gene transcription is negatively regulated in a cell-/tissue-specific manner by the YY1 transcription factor, which may form the complex with gene silencing factors, including HDACs, on the −183 to −137 promoter region of *Stx1a*. In addition, a genetic deficiency of YY1 involved in *Stx1a* transcription has been reported to cause an autosomal dominant neurodevelopmental disorder, Gabriele–DeVries syndrome; this disorder is characterized by neuronal symptoms, such as intellectual disability, variable cognitive impairment and behavioral problems [23]. Given that the *Stx1a* gene regulated by YY1 is known to be implicated in neuronal transmission [3,4], further elucidation of the regulatory mechanism of *Stx1a* expression will aid in understanding how YY1-regulated genes are controlled in the CNS and the role of the *Stx1a* promoter region in pathogenesis.

## Figures and Tables

**Table 1:** Mass spectrometry analyses data of protein (APP1) purified through Biotin-OL2 DNA beads. Histone deacetylase 1(HDAC1) highlighted with white text on black background.

Accession	Description	Score	Unique Peptides	PSMs
Q5FVM4	Non-POU domain-containing octamer-binding protein	7062.10	20	245
P61980	Heterogeneous nuclear ribonucleoprotein K	6418.99	18	252
Q4QQW4	Histone deacetylase 1	152.09	3	5
Q62733	Lamina-associated polypeptide 2, isoform beta	144.17	4	5
P09414	Nuclear factor 1A	76.54	2	2
Q6P747	Heterochromatin protein 1 binding protein 3	38.16	2	2

**Table 2:** Mass spectrometry analyses data of protein (APP2) purified through Biotin-OL2 DNA beads. Transcriptional repressor protein YY1 highlighted with white text on black background.

Accession	Description	Score	Unique Peptides	PSMs
27545350	Transcriptional repressor protein YY1	57.23	2	2
149065165	Nuclear respiratory factor 1 (predicted)	51.96	2	2

## Data Availability

The authors confirm that the data supporting the findings of this study are available within the article.
